# An experimental study: Does inbreeding increase the motivation to mate?

**DOI:** 10.1371/journal.pone.0199182

**Published:** 2018-06-18

**Authors:** Raïssa A. de Boer, Marcel Eens, Wendt Müller

**Affiliations:** Behavioural Ecology and Ecophysiology Group, University of Antwerp, Universiteitsplein 1, Wilrijk, Belgium; University of Missouri Columbia, UNITED STATES

## Abstract

Inbreeding is a central topic in evolutionary biology and ecology and is of major concern for the conservation of endangered species. Yet, it remains challenging to comprehend the fitness consequences of inbreeding, because studies typically focus only on short-term effects on inbreeding in the offspring (e.g. survival until independence). However, there is no a priori reason to assume that inbreeding has no more effects in adulthood. Specifically, inbred males should have lower reproductive success than outbred males among other things because of inbreeding depression in attractiveness to females and a reduced lifespan. Such differences in future reproductive value should affect male mating behaviour, such that an inbred male of a given age should be more motivated to seize a current mating opportunity than an outbred male of the same age. We used an inventive experimental set-up that enabled us to assess male behaviour in relation to an apparent mating opportunity while excluding potential confounding effects of female preference. Age-, weight-, and size-matched inbred and outbred male canaries (*Serinus canaria*) were presented with a female that only one male at a time could access visually via a ‘peephole’ and thus when both males were equally interested in seizing the apparent mating opportunity this would result in contest. We find that inbred males spent more than twice as much time ‘peeping’ at the female than outbred males, suggesting that inbreeding indeed causes different behavioural responses to an apparent mating opportunity. Our study is among the first to highlight that inbreeding affects male mating behaviour, and therewith potentially male-male competition, which should be taken into account in order to understand the full range of inbreeding effects on fitness.

## Introduction

Mating between related individuals often leads to negative effects on fitness (‘inbreeding depression’) [1], which has been shown in a wide variety of animal species [2]. The negative effects of inbreeding on fitness result in selection pressures which can affect reproductive behaviour and dispersal strategies among other things. Inbreeding depression is therefore a central topic in evolutionary biology and ecology [3,4]. Moreover, due to human-induced changes to the environment many populations become fragmented, which increases the occurrence of inbreeding and ultimately extinction risk. This further underscores the importance of understanding the potential fitness consequences of inbreeding [5,6]. Yet, research on inbreeding typically focuses on short-term effects of inbreeding in the offspring (e.g. survival until independence), which currently limits our understanding of the reproductive costs of inbreeding [7].

However, there is evidence that inbreeding may reduce lifetime reproductive success via a number of pathways. Inbreeding depression reduces adult lifespan [2,8,9], fecundity in females [10] and/or sperm performance in males [11–13]. Furthermore, male reproductive success, which is in large part determined by mating success, could also be negatively affected by inbreeding because inbred males are less attractive to females [14–19] or have more difficulty obtaining a territory [20–22] than outbred males. Inbreeding thus reduces the number of mating opportunities during the (presumably) shorter life of an inbred male and this should significantly increase the justified costs associated with a current mating opportunity. The fact that such asymmetries in future reproductive value can have far-reaching implications on reproductive strategies [23] is rarely considered.

A recent study on burying beetles (*Nicrophorus vespilloides*) showed that inbred males increased competitive effort in a current reproductive opportunity compared to outbred males. Inbred males were more risk-taking and were willing to suffer greater injuries while defending their brood [24]. Another study on prairie voles (*Microtus ochrogaster*) found that inbred males resided longer in the nest with the mother of their offspring than outbred males. Interestingly, this behavioural adaptation seemed to increase the fitness of their offspring. As a result, inbred and outbred males produced the same number of grand offspring even though inbred males produced fewer offspring than outbred males [9]. These findings show that a shift in behavioural strategy could ‘buffer’ the negative effects of inbreeding on male mating success.

Here, we test the hypothesis that inbreeding leads to differences in male mating behaviour. We examined the response of weight-, age-, and size-matched pairs of inbred and outbred male canaries (*Serinus canaria*) to a female (an apparent mating opportunity). In a unique experimental set-up we make use of a ‘peephole’ in which only one male at a time can obtain visual access to a female. Similar experimental set-ups that use small windows have been applied successfully in another bird species (Japanese quail; *Coturnix japonica*) [25–27]. This set-up enables us to score the males’ motivation to obtain access to the female while excluding female preference. In addition, when both males are equally motivated to seize an apparent mating opportunity this would elicit competition for the position in front of the peephole and thus contest. We expect to find that inbred males are more motivated to access the female because inbred males have lower future reproductive value than outbred males.

## Methods

### Study subjects

For this study 36 male canaries were used that hatched in the spring of 2013. Half of the focal males originated from full-sibling pairs (= inbred males), and half from unrelated pairs (= outbred males). The parental generation belonged to an outbred population kept at the University of Antwerp. Breeding cages (50 x 64 x 40 cm^3^, GEHU cages, the Netherlands) were equipped with shell sand, two perches, a nest cup, nesting material, ad libitum access to seeds (Van Camp, Belgium), and water. After the first chick had hatched, the parents additionally received egg food (Van Camp, Belgium) that was enriched with Orlux hand-mix (Versele-Laga) and freshly germinated seeds. At fledging (±25 days old) inbred and outbred males were matched into 18 pairs while controlling for body mass and size (see results) and age (N = 18, 0.6±0.3 days difference). In addition, we only used males that hatched first (day *i*) or second (day *i* + 1) in a brood because hatching order affected growth rate [28]. Each pair of males were housed from that time onwards together and additionally accompanied by an older, unrelated and unfamiliar male for song tutoring purposes [29]. The inbred/outbred pairs of males thus experienced the same environmental conditions from fledging until adulthood. Over this time course, the light regime was gradually changed from 14h light:10h dark to 10h light:14h dark and back again to mimic a seasonal cycle. The birds were tested in the staged male-male encounters in January 2014 after tutor males were removed from the cages and reproductive state was induced by exposure to a long light schedule for one month. The light schedule differed from natural seasonal light changes to allow the performance of consecutive breeding experiments in our facilities. All birds were weighed before the start of the experiments (December 2013).

### Experimental design

**Fig 1:**
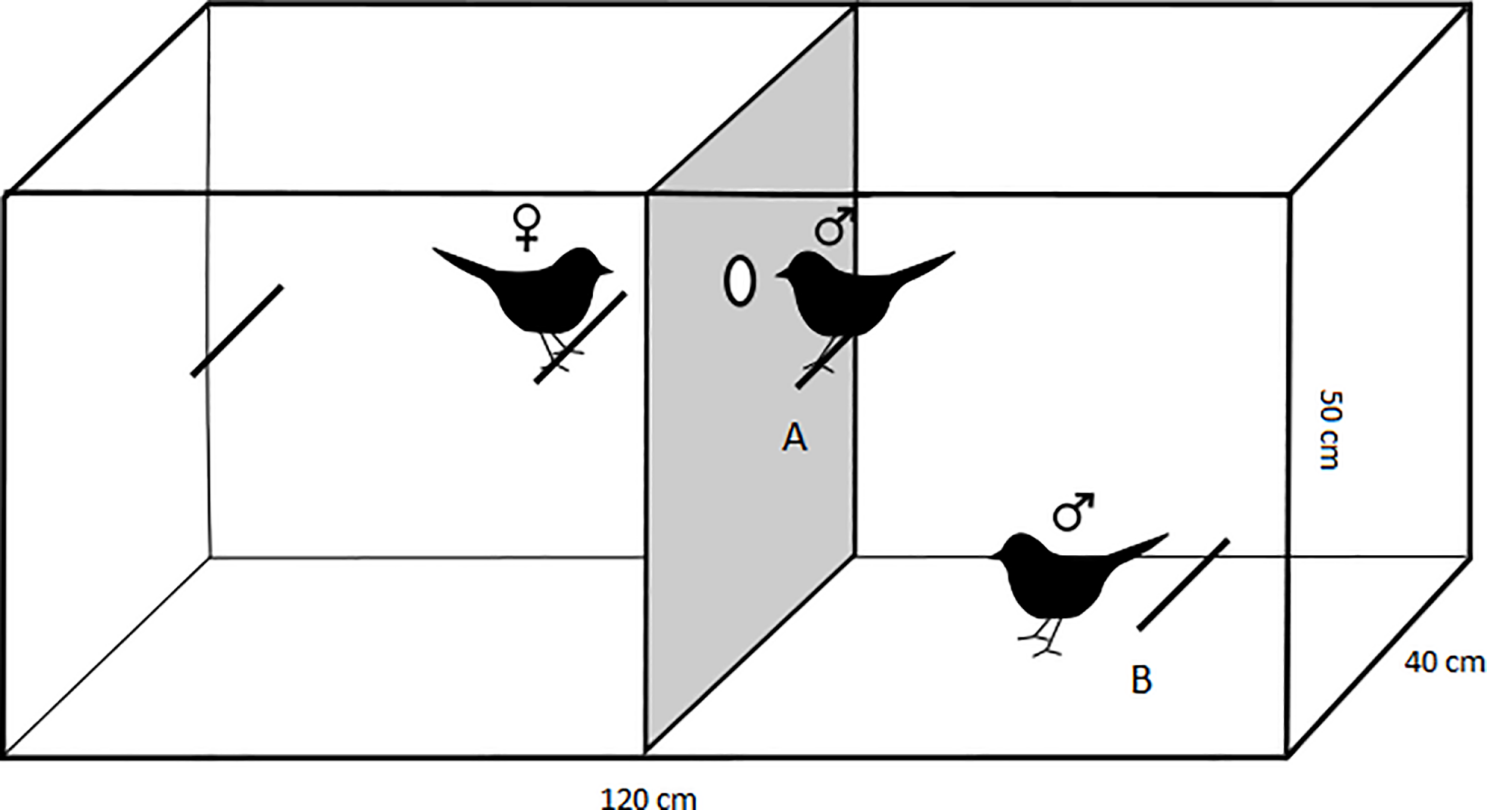
An experimental set-up using a peephole to assess the behaviour of inbred and outbred males in response to a mating opportunity. A cage was divided into two compartments using a cardboard wall that contained a small peephole. Males could only obtain visual access to the female on the other side of the cage by positioning themselves in front of the peephole on perch A. Only one male at the time could occupy this position, which could elicit competition. Perch B did not allow visual access to the female and served as a control position in the cage.

The experiments were set up in large cages (50 x 120 x 40 cm^3^) in which the inbred/outbred male pairs had been housed from fledging onwards. These home cages were divided into two compartments with a cardboard wall at the start of the experiments (Fig 1), with both compartments having access to seeds and water. Birds on either side of the cage could obtain visual access to the other side of the cage via a small (2 cm ∅) circle that was cut out of the cardboard (see for comparable set-up in quail [25–27]). The peephole was not large enough for a canary to fit through and birds could thus not move to the other side of the cage. An inbred/outbred pair of males (= dyad) that had been reared together was located on one side of the separation wall, and an outbred female that was unrelated to both males was then placed in the other compartment. It was alternated at which side of the cage the female or the males were placed and the setup of the cage was changed accordingly (Fig 1). The females originated from the same breeding season of the males and were thus the same age. All females were housed in large flight cages within the room and were within visual and acoustic access to the males during the entire period from fledging until adulthood and including the experiments. A different female was used for each of the 18 dyads.

Both sides of the cages contained two perches. On the side of the female both perches were placed at the level of the peephole to maximize the time spent time within easy visual access to the males. Females were not recorded and it was thus not analysed how much time females spent on each perch. On the side of the males, one perch was placed at the level of the peephole (= perch ‘A’, Fig 1), and the second perch was placed at the bottom of the cage that did not allow visual access to the peephole (= perch ‘B’, Fig 1). Thus, males could only obtain visual access to the female by positioning themselves in front of the peephole while sitting on perch A. This could elicit competition between the males, because although two males could sit on perch A, only one male at a time could occupy the position in front of the peephole to look at the female. Perch B served as a standardized control position in the cage because visual access to the female was impossible from that position in the cage thus access to this perch should not elicit competition.

After a female was placed in the experimental set-up the males were video-recorded for one hour. From the video recordings it was analysed with The Observer (Noldus) how much time each male spent peeping at the female, and in addition we counted the occurrence of various parameters of behaviour that were considered important in determining aggression (attack, chase, threat display), avoidance (escape, move away), or displacement behaviour (beak wipe, feather shuffle, preening) (Table 1). It was also noted how much time each bird spent on perch A and on perch B.

**Table 1:** A description of the behavioural parameters that were scored during the time inbred and outbred males were exposed to a female behind a peephole.

Behaviour	Description
Attack	Pecking and using feet to (attempt to) pin the opponent down
Beak wipes	Rubbing the bill along a substrate repetitively
Chase	Chasing the opponent in flight
Escape	Escape from a chase or attack by flying away from the opponent
Feather shuffle	Briefly pilo-erecting the feathers with a shuffling movement
Move away	Stepping away from the opponent without flying away
Preening	Scratching and/or cleaning the feathers
Threat display	Staring at the opponent with the wings spread out and the head lowered
Time spent peeping	Time spent in front of the peephole and looking at the female
Time spent on perch A	Time spent on the perch that gives visual access to the peephole
Time spent on perch B	Time spent on the perch that does not give visual access to the peephole

### Statistical analysis

In order to perform the main statistical analysis in which we tested the effect of inbreeding on behaviour in the experimental set-up we performed two prior analyses. The point of these analyses was to test (1) whether the behavioural parameters indeed grouped together into predefined categories of behaviour and (2) whether body mass was still similar between inbred and outbred birds within dyads so that this could not affect the outcome of the experiments, because birds were matched for body mass at fledging while the experiments were performed in adulthood.

To test (1) whether the different behavioural parameters grouped together into predefined different categories of behaviour a principal component analysis (PCA) was performed using the function ‘prcomp’ in the R package stats version 3.4.3. The different parameters were scaled and centered prior to the analysis. Overt attacks were excluded from the analysis because this was only observed in three birds, and including this parameter was thus not considered to be an accurate representation of male behaviour in this study. Principal components that had eigenvalues larger than 1 were retained, and parameters that had a factor loading larger than 0.40 were considered to be an important parameter for that principal component. The PCA resulted in three principal components (PC’s), reflecting three different behavioural categories (displacement, avoidance and aggressive behaviour; see results).

To test (2) whether there were indeed no differences in body mass and size between inbred and outbred birds within each dyad we used a multivariate Bayesian approach with Markov chain Monte Carlo (MCMC) algorithms implemented in the *MCMCglmm* R package [30]. The response variables were body mass at fledging, body mass at the start of the experiments and tarsus length (measured at fledging). The variables were scaled prior to analysis. Inbreeding status was included as a fixed effect. Non-independent measurements of the paired design were corrected for by including a random effect of dyad identity.

The main analysis was performed using another MCMC analysis in which we tested the effect of inbreeding on behaviour in response to a mating opportunity. The response variables included in the model were the PC scores (avoidance, displacement, aggression), time spent peeping, total time spent on perch A, and the total time spent on perch B. All response variables had a Gaussian error distribution. The PC scores of avoidance and aggression were multiplied by -1 so that higher scores represented more avoidance or aggression for the ease of interpretation. All response variables were scaled prior to the analysis. We included inbreeding as a fixed effect. In addition, we assessed the effect of body mass and size (= body condition) on behaviour during the experiment. To acquire a composite measure of body condition we used the scaled mass index which is the body mass standardized for size (see details in [31]). This continuous measure is based on tarsus length and body mass (measured before the experiments) under the assumption that tarsus length at fledging is a stable measure of body size (e.g. [32–34]). We scaled and centered the composite measure of body size for ease of interpretation. Non-independent measurements of our paired design were corrected for by including a random effect of dyad identity.

Both MCMC chains were run for 1100000 iterations with a burn-in phase of 100000 iterations, and 1000 independent samples were taken from the posterior at intervals of 1000 iterations. Convergence was determined by visual inspection of the traces and autocorrelation plots. The results of the MCMC algorithms are presented as the estimates of the sampled iterations with a 95% confidence interval. Statistical significance of the estimate can be assumed when confidence intervals do not overlap with zero. R software [35] was used for all analyses. Results are presented as mean ± SEM.

### Ethics statement

Our study has been carried out according to the relevant Belgian rules and guidelines and the above described experiments have been approved by the University of Antwerp ethical committee (file number 2011–86).

## Results

The PCA analysis resulted in three principal components that had eigenvalues larger than 1, which together explained 69% of the total variance (Table 2). The first component, accounting for 29% of the total variance, was defined by the occurrence of beak wipes, preening and feather shuffles. This PC was interpreted as displacement behaviour. The second component, explaining 24% of the total variance, was primarily loaded with the occurrence of escapes and moving away from the opponent (= avoidance behaviour). The last component, that was responsible for 17% of the variance, was defined by the occurrence of chases and threat displays (= aggressive behaviour).

**Table 2:** The results of the PCA analysis in order to group behavioural parameters that were observed during the time inbred and outbred males could obtain visual access to a female by positioning themselves in front of a peephole. PC1 was interpreted as displacement behaviour, PC2 as avoidance behaviour and PC3 as aggressive behaviour.

	PC1	PC2	PC3
Eigenvalue	1.418	1.285	1.074
Proportion of total variance	0.287	0.236	0.165
Cumulative Proportion	0.287	0.523	0.688
Beak wipes	**-0.472**	0.155	-0.268
Chase	0.372	0.196	**-0.524**
Escape	0.146	**0.638**	0.263
Feather shuffle	**-0.500**	0.171	-0.130
Move away	0.085	**0.616**	0.341
Preening	**-0.539**	0.236	-0.194
Threat display	0.262	0.258	**-0.643**

Inbreeding status did not affect tarsus length (0.41 [-0.12; 1.01], *P* = 0.15) (inbred: N = 18, 17.9±0.2 mm, outbred: N = 18, 18.2±0.1 mm), body mass at fledging (0.09 [-0.12; 0.30], *P* = 0.38) (inbred: N = 18, 18.7±0.3 g, outbred: N = 18, 18.8±0.3 g) and body mass before the experiments (0.004 [-0.66; 0.59], *P*>0.99) (inbred: N = 18, 23.7±0.6 g, outbred: N = 18, 23.6±0.6 g). Thus, inbred and outbred birds were successfully paired in dyads that were matched for body size and mass and also did not differ in body condition at the start of the experiments.

**Fig 2:**
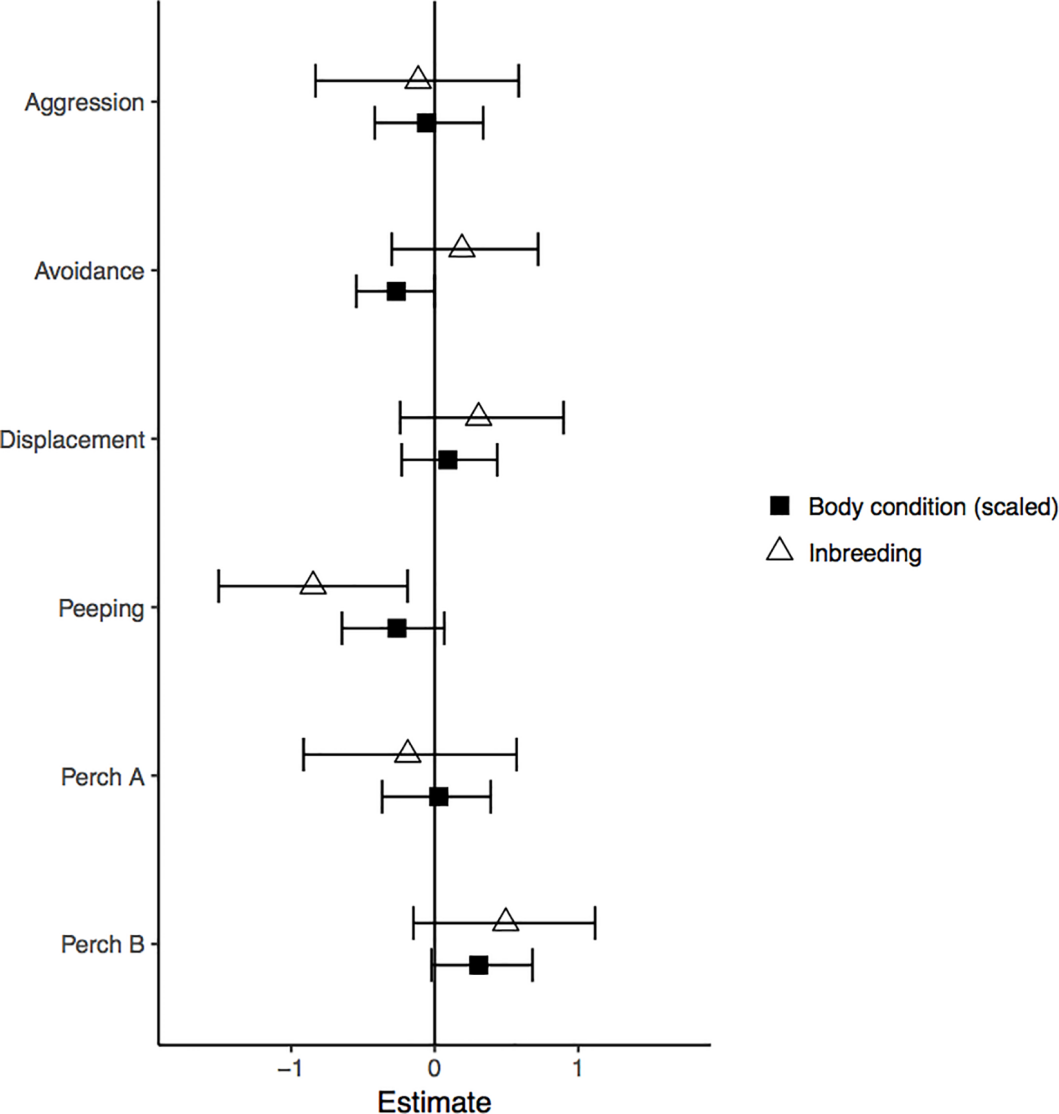
Parameter estimates with confidence intervals of Markov chain Monte Carlo generalized linear mixed models. The effects of inbreeding and body condition on aggression, avoidance and displacement behaviour, and on the time spent peeping, the total time spent on perch A and on perch B. The time spent peeping was significantly affected by inbreeding as shown by the confidence interval not overlapping with zero.

**Fig 3:**
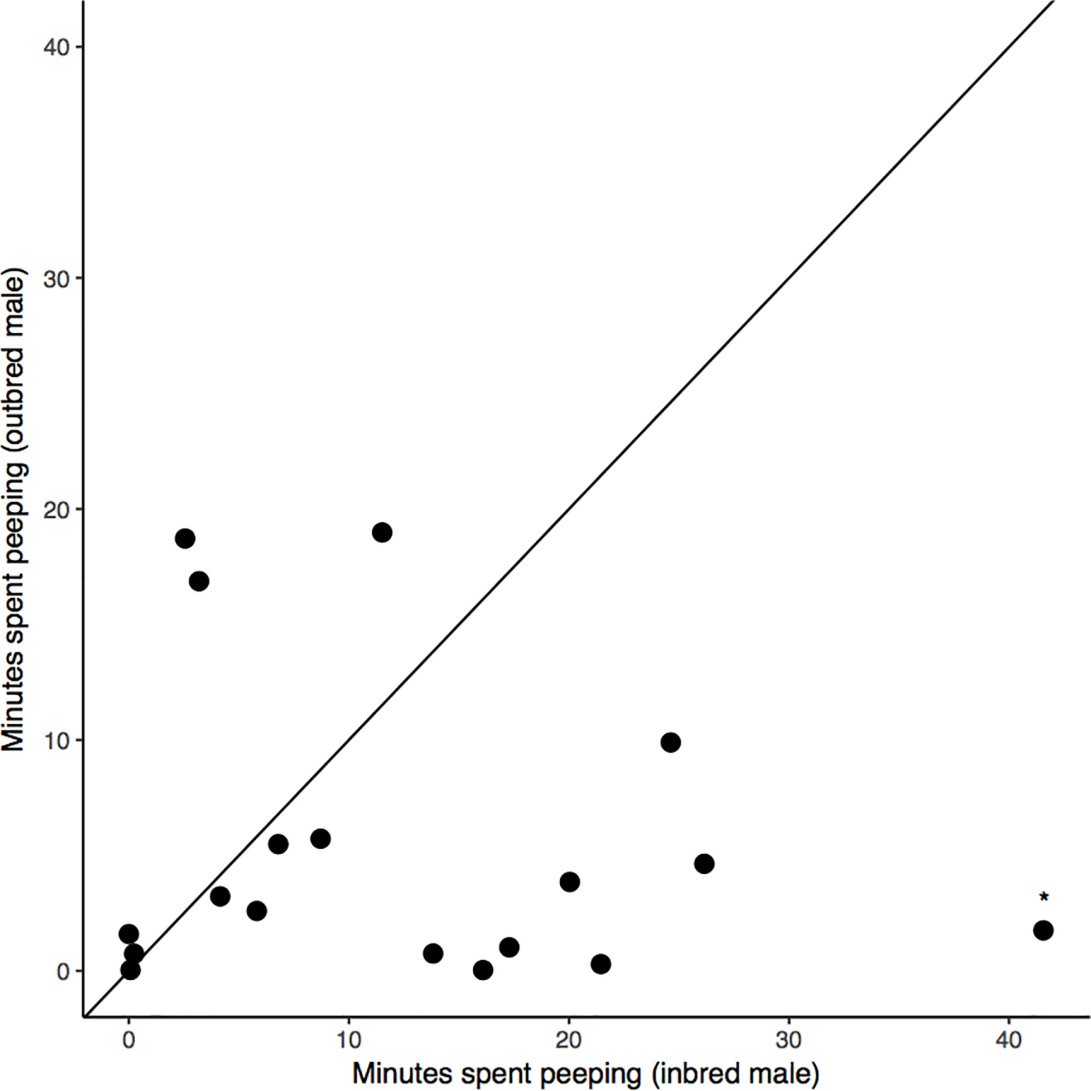
Peeping behaviour of inbred and outbred males with each data point representing a dyad of an inbred and an outbred male. During 60 minutes of being exposed to a female that was accessible visually by perching in front of peephole, inbred males positioned themselves significantly more time in front of the peephole to peep at a female on the other side of the cage than their outbred opponents. This result remained significant after rerunning the analysis without the dyad indicated with a star.

The MCMC analyses revealed that the amount of time individuals spent peeping depended on inbreeding status (-0.85 [-1.51; -0.19], *P* = 0.01; Fig 2). Inbred males (N = 18, 12.5±2.7 min) spent on average more time peeping through the peephole than their outbred counterparts (N = 18, 5.3±1.5 min) (Fig 3). This result was robust and remained significant (-0.64 [-1.2; -0.09], *P* = 0.03) after excluding a potential outlying dyad (indicated in Fig 3). The total time spent on perch A did not differ significantly between inbred and outbred males (-0.19 [-0.91;0.57], *P* = 0.6; Fig 2) (inbred: N = 18, 26.12±3.6 min, outbred: N = 18, 17.4±3.4 min), neither did the time spent on perch B (0.49 [-0.15;1.12], *P* = 0.12; Fig 2) (inbred: N = 18, 10.6±1.4 min, outbred: N = 18, 12.9±1.5 min). Of the total time spent on perch A, inbred birds spent 41±6% of the time peeping at the female, whereas outbred birds allocated 28±5% of the time spent on perch A to peeping at the female. Body condition did not significantly affect the time spent peeping (-0.26 [-0.65;0.07], *P* = 0.14), and the time spent on perch A (0.03 [-0.37;0.39], *P* = 0.89) and on perch B (0.31 [-0.02;0.68], *P* = 0.08) (Fig 2).

Inbreeding status did not affect the occurrence of avoidance (0.19 [-0.30;0.72], *P* = 0.46), displacement (0.31 [-0.24;0.90], *P* = 0.29), and aggressive (-0.12 [-0.83;0.58], *P* = 0.75) behaviour (Fig 2). Body condition tended to affect avoidance behaviour (-0.27 [-0.55; -0.002], *P* = 0.05; Fig 2), with males having lower body condition indexes showing relatively more avoidance behaviour than males with higher body condition indexes. Body condition did not affect the occurrence of displacement (0.09 [-0.23;0.44], *P* = 0.59) and aggressive (-0.06 [-0.42;0.34], *P* = 0.73) behaviour (Fig 2).

**Fig 4:**
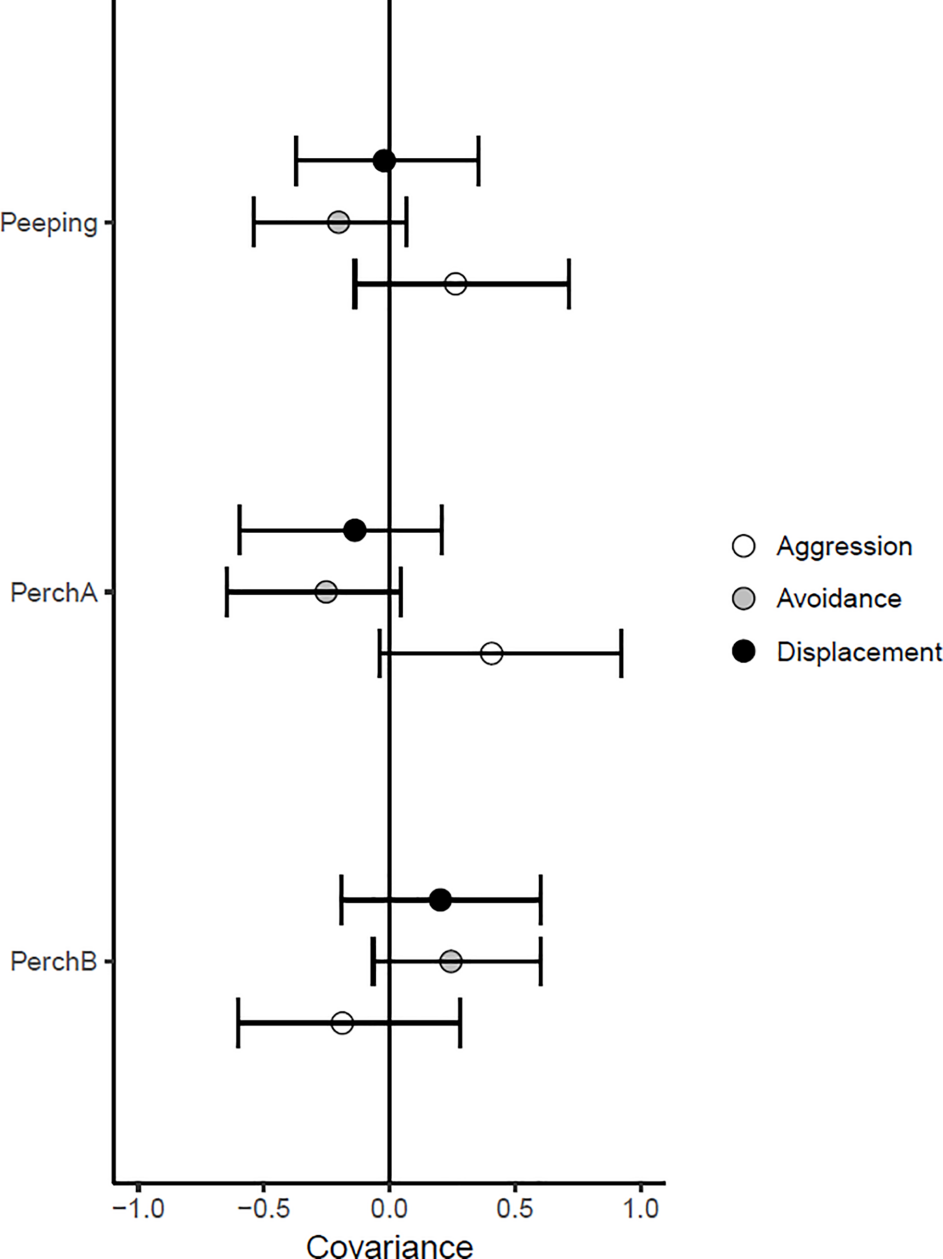
**The covariation of aggression, avoidance and displacement behaviour with the time spent peeping, the time spent on perch A and the time spent on perch B.** The PC scores of avoidance and aggression were multiplied by -1 for easier interpretation of the covariance. The time spent peeping and the total amount of time males spent on perch A, where birds could position themselves in front of the peephole to look at a female, tended to show positive covariation with aggression, and negative covariation with avoidance. The time birds spent on perch B, a control position in the cage that did not enable visual access to a female, tended to show positive covariation with the expression of behaviours indicative of avoidance.

The amount of time males spent peeping, and the total amount of time males spent on perch A covaried positively with the occurrence of aggression, and negatively with the occurrence of avoidance behaviour although confidence intervals overlapped with zero (Fig 4). On the other hand, the time spent on perch B which did not give visual access to the female tended to covary positively with avoidance behaviour (Fig 4). Furthermore, males that showed more avoidance behaviour also showed more displacement behaviour as indicated by positive covariation between these variables (0.42 [0.14; 0.76]). Last, the display of aggressive behaviour showed a negative covariation with avoidance behaviour (-0.41 [-0.79; -0.04]).

## Discussion

In this study we examined the behaviour of inbred and outbred males while exposed to an apparent mating opportunity and rated the time each male occupied the position in front of a peephole to look at a female. We have previously shown that inbreeding in these birds led to negative effects on growth [28], song expression and attractiveness [29]. Here, we show that inbred males spent on average twice the amount of time peeping at the female compared to outbred males. In line with our hypothesis, this may suggest that inbreeding causes a behavioural change with inbred males having a higher motivation to seize an apparent mating opportunity than outbred males. These differences in mating and reproductive behaviour could be due to differences in future reproductive value [9,24], which is expected to be lower in inbred than in outbred males.

The time spent on the perch that was enabling visual access to the female was associated with aggressive behaviour, suggesting that this perch was a preferred position in the cage. Indeed, in our set-up only one male at a time could sit in front of and look through the peephole and when both birds were interested in peeping at the female this would result in contest. The time spent on the control perch then again was associated with avoidance behaviour. The occurrence of aggression and avoidance suggests that there occurred some contest over the position in front of the peephole. This, in turn, implies that inbred males spent more time peeping at the female because outbred males yielded the position in front of the peephole to the inbred males rather than that they lacked interest in the apparent mating opportunity.

Intriguingly, aggressive behaviour was shown equally often by inbred and outbred males, thus outbred males did not forego peeping at the female as a result of a difference in the occurrence of aggressive behaviour. A higher motivation of inbred males to seize the apparent mating opportunity than outbred males could nevertheless cause them to be more risk-taking and willing to escalate into overt aggression [24]. This could have been communicated to the outbred males via intentional signaling [36], however, this remains speculative. The finding that there was no difference in the occurrence of aggression between inbred and outbred males somewhat contradicts previous findings that show that inbred males are worse competitors or less aggressive than outbred males [22,37–40]. This discrepancy might be explained by our experimental design in which we carefully controlled for differences in weight, size, and age between inbred and outbred males that could potentially mask inbreeding-induced differences in behaviour, whereas in previous studies this is rarely controlled for (but see [24]).

An alternative interpretation of our findings is that regardless of the presence of a female, males preferred the higher positioned perch in the cage because birds often prefer higher positions as has been found in zebra finches [41] and starlings [42]. Unfortunately, we have not tested such preference for high perches without a female present. However, a large proportion (28% outbred males, 41% inbred males) of the total time on the higher perch was spent with peeping, suggesting this perch was preferred in order to be able to peep at the female and not merely for obtaining a high position within the cage. In addition, as opposed to peeping at the female both birds could sit on the high perch at the same time and this should therefore not result in contest. We did not record the amount of time two males shared a perch during the experiments, but in other contexts where canaries share cages two birds are observed sharing a perch regularly (personal observation).

An obvious limitation of our set-up is that we used a rather artificial way of examining mating behaviour. The main aim of the set-up was to evaluate male behaviour in response to a female’s presence (i.e. mating opportunity) while excluding potential effects of female preference by not allowing physical access to the female. In the current study males were housed with females present in large flight cages within the room. Yet, males clearly became aroused and more active after a female was presented at the other side of the cage compared to the prior housing conditions where no female was present within the cage (personal observation). This indicates that the set-up is well suited for the aims of our study. Furthermore, similar set-ups have been used successfully in Japanese quail (*Coturnix japonica*), in which sitting in front of a window behind which a female was presented was a clear indication of sexual arousal [25–27].

Unfortunately, we could not elucidate whether the inbred or outbred male would have seized the mating opportunity and thus how an increased motivation would affect mating success. It could be that this results in alternative mating strategies. For example, a previous study on guppies (*Poecilia reticulata*) showed that there was no inbreeding depression in gonopodiual thrusting, a ‘sneaky’ mating strategy that circumvents female mate choice. Yet, the more costly mating strategy where males court females with ‘sigmoid’ displays was significantly reduced by inbreeding [15,16]. This suggests that inbred males adjust their behavioural strategy to make ‘the best of a bad job’ [16]. Indeed, there are additional examples of behavioural adaptations that could mitigate the negative effects of inbreeding. For example, inbred parents (partially) compensate by increasing their parental care behaviour [43,44]. Likewise, inbred prairie voles compensated negative effects of inbreeding on reproductive success by staying longer in the nest with the mother of their offspring [9].

In conclusion, we suggest that inbreeding and the associated reduction in future reproductive value could potentially lead to differences in mating behaviour and increase the motivation to seize current mating opportunities. Behavioural adaptations may thus mitigate inbreeding depression but may only become evident when considering long-term effects of inbreeding on fitness.

## Supporting information

S1 File PCA. This file contains the occurrence of various behaviours of inbred and outbred male canaries in response to an apparent mating opportunity.
(CSV)

S2 File MCMC. This file contains the time spent peeping and the time spent on perch A and B in the experimental cage, scores of three principal components, and weight and size of inbred and outbred male canaries.
(CSV)
